# Optimizing the Surface Quality of L-PBF Ti6Al4V ELI Alloy via Electropolishing and Its Effect on Corrosion Resistance for Dental Applications

**DOI:** 10.1055/s-0045-1802572

**Published:** 2025-03-12

**Authors:** Venus Chatpaiboonwat, Vorapat Trachoo, Patcharapit Promoppatum, Viriyah Chobaomsup, Kanokwan Saengkiettiyut, Viritpon Srimaneepong, Kamolporn Wattanasirmkit

**Affiliations:** 1 Department of Prosthodontics, Faculty of Dentistry, Chulalongkorn University, Bangkok, Thailand; 2 Department of Oral and Maxillofacial Surgery, Faculty of Dentistry, Chulalongkorn University, Bangkok, Thailand; 3 Department of Mechanical Engineering, Faculty of Engineering, King Mongkut's University of Technology Thonburi, Bangkok, Thailand; 4 Metallurgy and Materials Science Research Institute (MMRI), Chulalongkorn University, Bangkok, Thailand

**Keywords:** corrosion, electropolishing, laser powder bed fusion, surface roughness, Ti6Al4V

## Abstract

**Objectives:**

The Ti6Al4V ELI alloy produced via laser powder bed fusion (L-PBF) has attracted interest for use in dental applications. However, surface finishing is an important property that can be managed by various methods. The purpose of this study was to investigate the effects of electropolishing (EP) on the surface roughness and corrosion resistance of L-PBF Ti6Al4V ELI alloy.

**Materials and Methods:**

The present study explored the influence of current density (0.3 A/cm ^2^ ), voltage (15 V), and distance (2 and 4 cm) on the surface quality of L-PBF-printed Ti6Al4V ELI. The potentiodynamic polarization testing was performed to investigate the corrosion behavior of electropolished Ti6Al4V ELI alloy plates.

**Statistical Analysis:**

The data variation was compared at different conditions of EP using a one-way analysis of variance and Tukey's *post hoc* testing at a significance level of 5%.

**Results:**

This study showed that EP significantly reduced the surface roughness and enhanced corrosion resistance of printed Ti6Al4V ELI alloy with the best result achieved by using 15 V and 2 cm of anode–cathode distance.

**Conclusion:**

This study indicates that customized EP settings are crucial for optimizing the surface properties of Ti6Al4V ELI for use in dental and biomedical applications. However, the corrosion resistance can be reduced due to increased porosity resulting from the EP treatment.

## Introduction

Traditional manufacturing methods like casting, forging, and machining have been the general methods for shaping metals into finished products. However, due to the limitations of these conventional methods such as material waste, design limit, and production time, additive manufacturing (AM) has drawn increased attention. The AM, also known as direct digital manufacturing, autonomous manufacturing, or three-dimensional (3D) printing, has been manufacturing many kinds of metals, including stainless steel, cobalt-chromium (CoCr), or titanium (Ti), for biomedical, aerospace, defense, and automotive industries. AM has rapidly risen as a popular technology because it enables the creation of complex geometric parts from digital models by adding material layer by layer. By providing on-demand production and reducing waste, expense, energy consumption, and carbon dioxide emissions, AM technology has the potential to completely change logistics and manufacturing operations. 1 One of the most widely utilized AM methods in dentistry is laser powder bed fusion (L-PBF).

In dentistry, AM enables digital workflows that combine intraoral scanning and advanced manufacturing processes to create metal prostheses for either fixed prostheses or removable dentures and also to facilitate the fabrication of implant-supported prostheses by integrating digital and conventional methods. 2 CoCr and Ti/ Ti alloys are two most frequently utilized metal alloys in AM technology in dentistry. 2 Ti6Al4V ELI alloy, also known as Ti grade 23, is a form of Ti6Al4V alloy (Ti grade 5), which is appropriate for dental implants and prostheses due to its strong corrosion resistance and constant biocompatibility, particularly in oral conditions. Furthermore, its mechanical properties make it ideal for applications requiring a strong and lightweight material. 3 4 5 L-PBF is commonly used for printing metal prostheses in dentistry. L-PBF operates by employing a laser beam to fuse metal powder incrementally layer by layer until the intended form is achieved. 6 Therefore, the surface finish of the printed parts is one of the primary concerns of this method. However, the as-printed surface roughness is a significant barrier to L-PBF applications. This is due to the surface roughness produced during L-PBF process, which can be rougher than that in conventional manufacturing procedures depending on the manufacturing parameters. 7 8

To resolve this drawback of surface roughness caused by the printing process, several methods have been introduced to improve the surface quality of 3D-printed parts, such as mechanical, chemical, or electrochemical surface treatments, laser-based surface treatments, and coatings. 9 These methods can compensate for technological deficiencies and mishaps, such as the staircase effect related to layer-by-layer deposition, partially melted powders, spatters, the balling effect, imprecise support removal, uncontrolled wetting, and melt pool instability. 7 8 These are also effective methods to modify the surfaces of L-PBF components. 9 10 11 Electropolishing (EP) is a method that removes the surface layer of the metal, and EP can improve the surface smoothness and generate a stable oxide film that enhances the corrosion resistance of Ti. 12 13 14 The major advantages of EP include eliminating surface imperfections and working with complex surface topography. 4 12 13 Zhang 14 investigated the electropolished Ti6Al4V alloy and found that the mean roughness (Ra) decreased, resulting in a smoother surface and improved corrosion resistance. Compared with other polishing methods, the EP is a promising polishing method for improving the surface quality of L-PBF components because of its simplicity and ability to polish complex structures effectively. 12

However, the EP also has limitations, mainly due to the complexity of the process, which is influenced by various parameters that can affect biophysical outcomes, such as the composition of the electrolyte, the distance between the anode and cathode, the shapes and areas of the anode and cathode, the agitation speed of the solution, temperature, duration, and the voltage or electric current used. 15 16 Therefore, it is a challenge to determine the optimal parameters suitable for each metal material. Although a previous study on the EP of L-PBF Ti6Al4V has focused on varying polishing durations and electrolyte solutions, a relationship between surface roughness after the EP and corrosion resistance remains unclear. 14 Therefore, this study aimed to investigate the effects of current density, voltage, and distances between anode and cathode of the EP method on the surface roughness and corrosion resistance of electropolished L-PBF Ti6Al4V ELI alloy.

## Materials and Methods

### Sample Preparation

Ti6Al4V ELI alloy plates sized 8 × 20 × 2 mm were fabricated using an L-PBF printing machine (TruPrint 1000, TRUMPF, Germany) with the printing parameters listed in Table 1 . Ti6Al4V ELI powder with a particle size distribution of 15 to 45 μm (AP&C GE Additive, Canada) was used as the base material.

**Table 1:** Process parameters of L-PBF manufacturing used to fabricate the samples

Process parameters	Value
Laser power (W)	125
Scanning speed (mm/s)	1200
Layer thickness (μm)	20
Hatch spacing (μm)	110
Laser spot size (μm)	30
Scanning pattern	Chess

Abbreviation: L-PBF, laser powder bed fusion.

### Procedure of EP

**Fig. 1:**
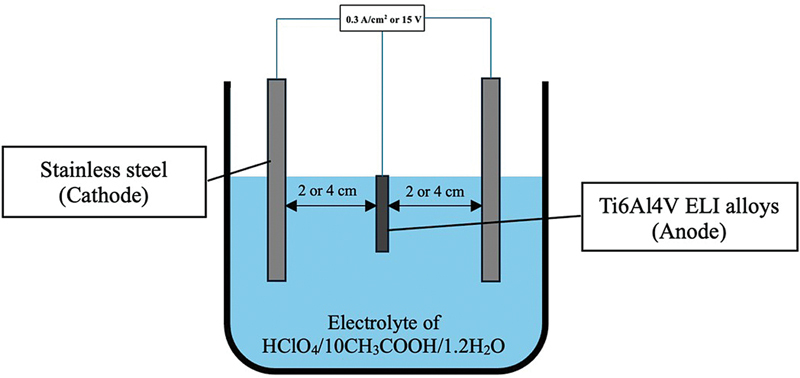
Schematic diagram of the electropolishing process.

Before conducting the EP, all Ti6Al4V ELI alloy plates were ultrasonically washed with acetone for 15 minutes in order to get rid of the remaining metal powder from their surfaces but no chemical or mechanical surface polishing was applied to the samples. The Ti6Al4V ELI alloy plates were electropolished in an electrolyte solution of perchloric acid (HClO _4_ ), glacial acetic acid (CH _3_ COOH), and distilled water (H2O) in a volume ratio of 1:10:1.2 for 15 minutes at room temperature. 14 The cathode was stainless steel and the anode was a Ti6Al4V ELI printed plate ( Fig. 1 ). Based on the parameters of EP, the Ti6Al4V ELI alloy samples were divided into five groups (with group labels), as indicated in Table 2 . Note that 0.3/2 and 0.3/4 EP groups were performed at a current density of 0.3 A/cm ^2^ with 2 and 4 cm distances between anode–cathode distances. Note that 15/2 and 15/4 EP groups were conducted with a voltage of 15 V, and the distances between the anode and cathode were 2 and 4 cm, respectively. During the EP, the electrolyte was stirred at 500 revolutions per minute using a magnetic stirrer to remove the remaining reactants from the surface. After EP, all specimens were cleaned with acetone for 15 minutes using an ultrasonic cleaner.

**Table 2:** Setup parameters of electropolishing (EP) for each experimental group

Experimental group	Condition of the EP process	Anode–cathode distance (cm)	Group label
Control	N/A	N/A	Control
1	0.3 A/cm ^2^	2	0.3/2
2		4	0.3/4
3	15 V	2	15/2
4		4	15/4

Abbreviation: N/A, not available.

### Surface Roughness and Surface Morphology

The surface roughness of the samples was determined using a contact profilometer (form Talysurf i-Series PRO, Taylor Hobson, United Kingdom). The surface roughness was measured using a stylus gage, configured at 1.040-mm ranges, with a speed of 0.1 mm/s, and length of 4 mm, three times per sample. The data were analyzed using Metrology 4.0 Software (Taylor Hobson, United Kingdom). The surface roughness values were represented as the arithmetic mean deviation (Ra), and four samples from each group were measured. The surface morphology was observed under a scanning electron microscope (Quanta250, FEI, United States).

### Corrosion Resistance Analysis

The corrosion resistance of the electropolished samples was assessed by potentiodynamic polarization testing using a standard three-electrode system. The Ti6Al4V ELI alloy plates with an area of 1 cm ^2^ were mounted to a working electrode, where a platinum electrode was used as the counter electrode and a saturated calomel electrode was used as the reference electrode. All measurements were performed in 3.5% NaCl solution at 27 °C using the μAutolab electrochemical workstation (μAutolab Type III, Eco Chemie, Utrecht, the Netherlands) operated by Autolab NOVA software version 1.11.2. The polarization curves were obtained from Tafel plots, and the waiting time for the stable open circuit potential (OCP) was set to be 600 seconds. The potential was then swept from –0.25 to +0.75 V versus the OCP at a scan rate of 1 mV/s while recording the corresponding current response. Tafel extrapolation was used to determine the corrosion potential ( *E*_corr_ ) and corrosion current density ( *I*_corr_ ) by examining the linear regions of the anodic and cathodic branches of the polarization curves. These parameters were then used to calculate the corrosion rate.

### Statistical Analysis

The surface roughness data were analyzed using SPSS version 22.0 (SPSS Inc., Chicago, Illinois, United States). The normality of data was determined using the Shapiro–Wilk test. A one-way analysis of variance and Tukey's *post hoc* tests were used to compare the surface roughness at various current densities, voltages, and anode–cathode distances. The significance level for all tests was set at 5%.

## Results

### Surface Roughness

The surface roughness values of the Ti6Al4V ELI printed samples for each group are listed in Table 3 . The surface roughness of all groups decreased after the EP. It was found that when using 15 V and a 2-cm distance, the surface roughness of the printed sample was the lowest ( *p* < 0.05). However, there was no significant difference among the other groups.

**Table 3:** Mean (SD) values of surface roughness of the Ti6Al4V ELI alloy experimental groups

Group label	Current density (A/cm ^2^ )	Voltage (V)	Anode–cathode distance (cm)	Ra (μm)
Control	–	–	–	6.28 (±0.65) ^a^
0.3/2	0.3	–	2	2.12 (±0.33) ^b^
0.3/4	0.3	–	4	2.19 (±0.34) ^b^
15/2	–	15	2	1.69 (±0.51) ^c^
15/4	–	15	4	1.95 (±0.29) ^bc^

Abbreviation: Ra, mean roughness; SD, standard deviation.

Note: Different superscript letters indicate significant differences between groups ( *p* < 0.05).

**Fig. 2:**
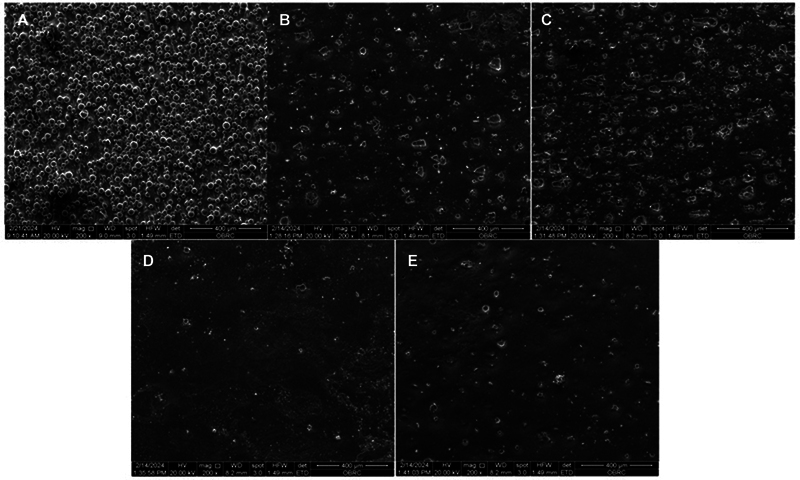
Scanning electron micrographs of the Ti6Al4V ELI printed samples: ( **A** ) as-printed, ( **B** ) 0.3/2, ( **C** ) 0.3/4, ( **D** ) 15/2, and ( **E** ) 15/4.

The surface morphologies of the specimens after the EP are shown in Fig. 2 . The surface morphology of as-printed Ti typically exhibits partially melted or unmelted powder particles adhering to the surface, and no powder particles were observed in the specimens after EP. Although the surface morphologies of the 15/2 EP group displayed lower surface roughness with comparatively smooth and homogeneous surface qualities, all groups showed porosity on the electropolish surfaces, regardless of EP condition.

### Corrosion Resistance

**Fig. 3:**
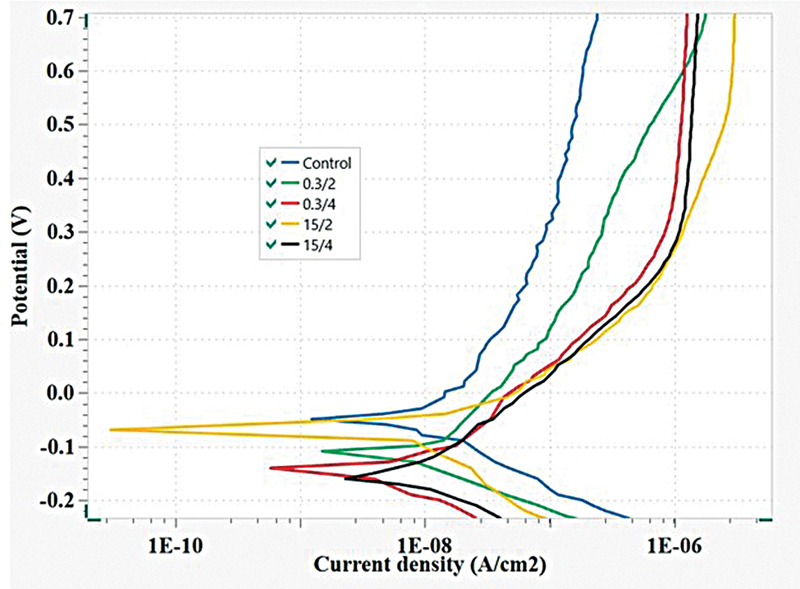
Representative potentiodynamic polarization curves.

The potentiodynamic curves exhibiting corrosion properties of the control and experimental groups are shown in Fig. 3 . The curves of the postprocessing specimens were comparable. The control group (as-printed) exhibited the highest *E*_corr_ , indicating superior corrosion resistance compared with the other electropolish groups. The control group's *I*_corr_ was relatively low, implying a minimum corrosion rate. This baseline performance highlights the inherent corrosion resistance of the Ti6Al4V ELI alloy in the absence of EP treatment, providing a reference point for evaluating the effects of various EP conditions.

The 15/2 EP sample presented an *E*_corr_ value closest to that of the control group. The *I*_corr_ value of this group was the lowest among all tested groups, implying the lowest corrosion rate. In contrast, the other condition groups had lower *E*_corr_ and higher *I*_corr_ than the 15/2 EP group. These results implied slightly higher corrosion rates. The increased porosity observed on the surfaces of these groups may have contributed to their reduced corrosion resistance. Despite the improvements in surface finishing, the porosity caused by the EP indicates that although the EP improved the surface quality, the reverse effect of the EP compromised the corrosion resistance. Additionally, the *I*_corr_ and corrosion rates of 0.3/2, 0.3/4, and 15/4 EP samples were similar to those of the control group ( Table 4 ).

**Table 4:** Corrosion parameter values of the Ti6Al4V ELI alloy groups

Sample group	*E*_corr_ [V]	*I*_corr_ [μA/cm ^2^ ]	Corrosion rate [mm/y]
Control	–0.045	0.016	0.00019
0.3/2	–0.126	0.018	0.00021
0.3/4	–0.146	0.011	0.00013
15/2	–0.068	0.008	0.00010
15/4	–0.150	0.015	0.00018

## Discussion

This study aimed to determine how various EP parameters, including current density, applied voltage, and anode–cathode distance, affect the corrosion resistance and surface roughness of L-PBF Ti6Al4V ELI alloy. Our findings showed that after the EP, the surface roughness of the L-PBF Ti6Al4V ELI alloy samples was consistently lower in all groups than in the control group. Notably, the group with voltage control exhibited lower surface roughness than the group with controlled current density. When evaluating the influence of the anode–cathode distance, no difference in surface roughness was observed between the groups. The results show that a shorter anode–cathode distance results in lower surface roughness. However, other factors would affect the electrochemical mechanism.

The surface roughness of as-printed Ti6Al4V ELI alloy sample is influenced by partially melted or unmelted powder particles adhering to the surface, resulting in a rough texture. This inherent roughness can compromise the material's mechanical properties and biocompatibility, especially in the field of medical applications. 17 The EP is a type of postprocessing treatment employed to improve the smoothness of alloy surfaces. The results showed that all EP samples exhibited a marked reduction in surface roughness compared with the as-printed samples, which is consistent with a study by Tsoeunyane et al. 18 Controlling the current density and voltage had a positive impact on the resulting surface roughness. A study by Urlea and Brailovski 19 demonstrated that controlling the current density significantly improved the surface roughness across all tested orientations and reduced Ra. Similarly, studies by Guo et al 20 on voltage control have shown a reduction in surface roughness.

The surface morphology revealed that all unmelted powder was completely removed by the EP. However, pitting was observed on the sample surfaces. This finding is consistent with the previous studies 18 21 reporting the use of an electrolyte containing perchloric acid (HClO _4_ ), as the chloride ions have considerable effect on the metal alloy. Moreover, Ti tetrachloride (TiCl _4_ ) films can cause uneven surfaces if not quickly removed. Optimal polishing occurs within a specific range of voltage but exceeds the limit of oxygen evolution and pitting. Proper stirring is essential to dissolve the TiCl _4_ film. The insufficient stirring can cause passivation, whereas excessive stirring results in uneven polishing and pitting. 22 23

In general, the EP smoothens surfaces by eliminating imperfections, thereby improving corrosion resistance by reducing the number of sites at which corrosion can start. Consequently, this typically leads to elevated *E*_corr_ . 17 However, despite the improvements in surface smoothness, the electropolished samples exhibited lower *E*_corr_ values. This phenomenon can be attributed to residual porosities that may remain on the surface layer after the EP. These residual pores can act as initiation sites for localized corrosion, leading to lower *E*_corr_ values when exposed during corrosion testing. 24 25 Similarly, Zhang et al 26 reported that the *E*_corr_ of Ti6Al4V ELI before polishing had a higher *E*_corr_ than after the EP. A decrease in *E*_corr_ represents lower thermodynamic stability, which indicates lower resistance of a material to chemical reactions with its environment. However, this metric alone does not capture all aspects of corrosion behavior, as other factors like kinetic barriers, protective films, and environmental conditions also play significant roles in determining the overall corrosion resistance. 27 28

When examining the *I*_corr_ and corrosion rate, the study found that they were not different between the groups, except for the 15/2 EP group, which had the lowest values. These results agree with the measured surface roughness, showing that the EP conditions at 15 V with 2-cm distance improved the surface roughness and enhanced the corrosion resistance. These findings indicate that surface imperfections affect corrosion performance and that the absence of porosity significantly enhances corrosion resistance. Similarly, Zhang 14 and Wu et al 29 evaluated the biocorrosion resistance of Ti6Al4V alloy after EP. Proper EP treatment can reduce surface roughness, resulting in a flatter surface with no defects and improved biocorrosion resistance.

Overall, this study emphasizes the importance of optimizing the EP parameters to maximize the performance and durability of Ti6Al4V ELI alloys, where both surface quality and corrosion resistance are crucial. The results showed that the EP with constant voltage produced better surface quality than constant current. However, different types of components or devices, such as 3D-printed dental prostheses or medical devices, may require adjustment of the EP parameters because these parameters cannot be universally applied. Additionally, this study identified the occurrence of pitting in some areas, indicating that combining the EP with other postprocessing treatments is needed to enhance surface performance, usability, and corrosion properties.

However, this study has the limitation that it focused on surface roughness as the key parameter influencing surface quality and its subsequent effect on corrosion resistance. While other surface properties such as surface hardness, wettability, or surface chemistry also play significant roles on surface quality. These factors should therefore be explored in future research to better understand their impact on corrosion resistance. In addition, the sample used in this study was restricted to a simple shape, which does not fully represent the complex geometries commonly found in dental or medical applications. The complex geometries may yield different outcomes due to their intricate surface geometry.

## Conclusion

EP significantly improved the surface roughness and morphology of the Ti6Al4V alloy. Interestingly, applying a constant voltage of 15 V with appropriate anode–cathode distance produced the lowest surface roughness and corrosion resistance. This could yield better results than employing the constant current. However, improper EP can reduce corrosion resistance by increasing surface porosity, which leads to pitting corrosion. These findings are essential for dental or medical applications, as surface quality and high corrosion resistance are crucial for the performance and longevity of prostheses. Consequently, appropriate EP can improve the L-PBF component's surface quality. This method could also be beneficial for surface finishing in other 3D-printing industries.
